# Supplementary material for: Impact of Maternal Malnutrition on Gut Barrier Defense: Implications for Pregnancy Health and Fetal Development
Source: Nutrients. 2019 Jun 19;11(6):1375. doi: 10.3390/nu11061375 (PMC6628366; doi:10.3390/nu11061375)
Supplement: Supplementary file 1 [file nutrients-11-01375-s001.pdf]

Supplementary Material

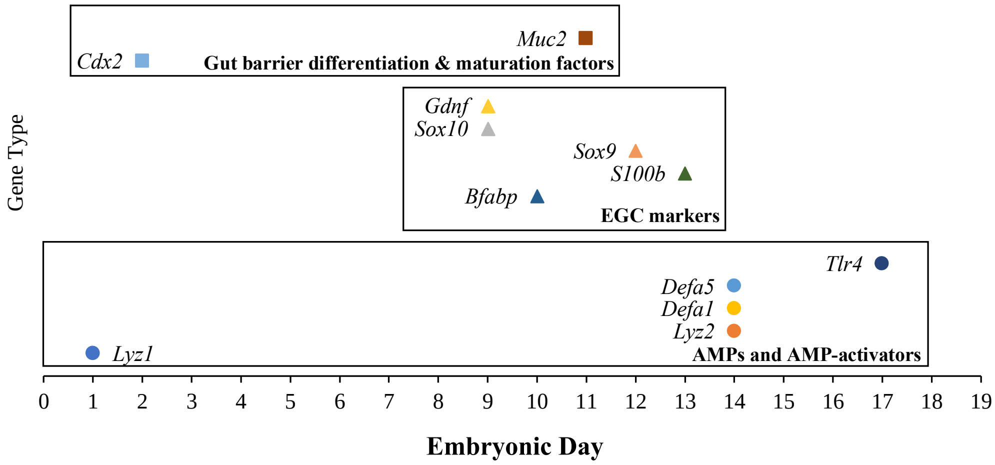

Figure S1. Earliest recorded expression of gut integrity, function, and development genes in the mouse fetal gut. Embryonic day of first established fetal gut expression of Paneth AMPs (*Lyz1, Lyz2, Defa1,* and *Defa5*), AMP-activator receptor (*Tlr4*), EGC markers (*Sox9, Sox10, S100b, Bfabp,* and *Gdnf*), gut barrier differentiation (*Cdx2*), and mucus production (*Muc2*) genes [1–9]. Genes are grouped by function. Fetal gut expression levels of *Pla2g2, Reg3g, Cldn-3, Cldn-7,* and *Plp1* could not be found in the literature.

Literature

1. Bry, L.; Falk, P.; Huttner, K.; Ouellette, A.; Midtvedt, T.; Gordon, J.I. Paneth cell differentiation in the developing intestine of normal and transgenic mice. *Proc. Natl. Acad. Sci. U. S. A.* **1994**, *91*, 10335–10339.
2. Young, H.M.; Bergner, A.J.; Müller, T. Acquisition of neuronal and glial markers by neural crest-derived cells in the mouse intestine. *J. Comp. Neurol.* **2003**, *456*, 1–11.
3. Burns, A.J.; Thapar, N. Advances in ontogeny of the enteric nervous system. *Neurogastroenterol. Motil. Off. J. Eur. Gastrointest. Motil. Soc.* **2006**, *18*, 876–887.
4. Filant, J.; Spencer, T.E. Cell-specific transcriptional profiling reveals candidate mechanisms regulating development and function of uterine epithelia in mice. *Biol. Reprod.* **2013**, *89*, 86.
5. Wulff, B.C.; Yu, L.; Parent, A.E.; Wilgus, T.A. Novel differences in the expression of inflammation-associated genes between mid- and late-gestational dermal fibroblasts. *Wound Repair Regen. Off. Publ. Wound Heal. Soc. Eur. Tissue Repair Soc.* **2013**, *21*, 103–112.
6. Good, M.; Siggers, R.H.; Sodhi, C.P.; Afrazi, A.; Alkhudari, F.; Egan, C.E.; Neal, M.D.; Yazji, I.; Jia, H.; Lin, J.; et al. Amniotic fluid inhibits Toll-like receptor 4 signaling in the fetal and neonatal intestinal epithelium. *Proc. Natl. Acad. Sci. U. S. A.* **2012**, *109*, 11330–11335.
7. Braga, V.M.; Pemberton, L.F.; Duhig, T.; Gendler, S.J. Spatial and temporal expression of an epithelial mucin, Muc-1, during mouse development. *Dev. Camb. Engl.* **1992**, *115*, 427–437.
8. Fu, L.; Shi, Y.-B. The Sox transcriptional factors: Functions during intestinal development in vertebrates. *Semin. Cell Dev. Biol.* **2017**, *63*, 58–67.

9. Beck, F.; Erler, T.; Russell, A.; James, R. Expression of Cdx-2 in the mouse embryo and placenta: possible role in patterning of the extra-embryonic membranes. *Dev. Dyn. Off. Publ. Am. Assoc. Anat.* **1995**, *204*, 219–227.
